# Conversion of deoxynivalenol to 3-acetyldeoxynivalenol in barley-derived fuel ethanol co-products with yeast expressing trichothecene 3-*O*-acetyltransferases

**DOI:** 10.1186/1754-6834-4-26

**Published:** 2011-09-02

**Authors:** Piyum A Khatibi, Justin Montanti, Nhuan P Nghiem, Kevin B Hicks, Greg Berger, Wynse S Brooks, Carl A Griffey, David G Schmale

**Affiliations:** 1 Virginia Tech, Department of Plant Pathology, Physiology and Weed Science, Blacksburg, VA 24061, Virginia Tech, USA; 2 Department of Crop and Soil Environmental Sciences, Blacksburg, VA 24061, USA; 3 Sustainable Biofuels and Co-Products Research Unit, USDA, ARS, Eastern Regional Research Center, Wyndmoor, PA 19038, USA

## Abstract

**Background:**

The trichothecene mycotoxin deoxynivalenol (DON) may be concentrated in distillers dried grains with solubles (DDGS; a co-product of fuel ethanol fermentation) when grain containing DON is used to produce fuel ethanol. Even low levels of DON (≤ 5 ppm) in DDGS sold as feed pose a significant threat to the health of monogastric animals. New and improved strategies to reduce DON in DDGS need to be developed and implemented to address this problem. Enzymes known as trichothecene 3-*O-*acetyltransferases convert DON to 3-acetyldeoxynivalenol (3ADON), and may reduce its toxicity in plants and animals.

**Results:**

Two *Fusarium *trichothecene 3-*O-*acetyltransferases (FgTRI101 and FfTRI201) were cloned and expressed in yeast (*Saccharomyces cerevisiae*) during a series of small-scale ethanol fermentations using barley (*Hordeum vulgare*). DON was concentrated 1.6 to 8.2 times in DDGS compared with the starting ground grain. During the fermentation process, FgTRI101 converted 9.2% to 55.3% of the DON to 3ADON, resulting in DDGS with reductions in DON and increases in 3ADON in the Virginia winter barley cultivars Eve, Thoroughbred and Price, and the experimental line VA06H-25. Analysis of barley mashes prepared from the barley line VA04B-125 showed that yeast expressing FfTRI201 were more effective at acetylating DON than those expressing FgTRI101; DON conversion for FfTRI201 ranged from 26.1% to 28.3%, whereas DON conversion for FgTRI101 ranged from 18.3% to 21.8% in VA04B-125 mashes. Ethanol yields were highest with the industrial yeast strain Ethanol Red^®^, which also consumed galactose when present in the mash.

**Conclusions:**

This study demonstrates the potential of using yeast expressing a trichothecene 3-*O*-acetyltransferase to modify DON during commercial fuel ethanol fermentation.

## Background

As the USA attempts to decrease its reliance on fossil fuels, alternative fuel sources are in high demand. Barley is an emerging alternative to corn as an important source for fuel ethanol [1]. Winter barley may be grown during the winter months, supplying an additional crop on land that would otherwise be fallow [2]. This would provide additional income for farmers, and an ethanol feedstock that does not compete with feed and food markets. In Virginia, new cultivars of barley with high starch content are being developed to support fuel ethanol production [3]. The USDA Agricultural Research Service predicts that North America will be able to produce up to 2 billion gallons of ethanol per year from barley alone [4,5].

A valuable co-product of fuel ethanol production, known as distillers dried grains with solubles (DDGS), is increasingly being used as a feed source for domestic animals [6]. DDGS contains high levels of protein, fiber, minerals and vitamins [7,8]. An increase in the supply and demand for DDGS [9] is expected to coincide with the increased production of fuel ethanol in commercial plants [10], which rely on the sale of DDGS to turn a profit [11].

One of the challenges facing the fuel ethanol industry is the management of mycotoxins such as deoxynivalenol (DON) in DDGS. Barley can become contaminated with DON in the field after infection with the fungal plant pathogen *Fusarium graminearum (teleomorph Gibberella zeae)*. Barley contaminated with high levels of DON is excluded from feeds and foods [12]. DON is a potent inhibitor of protein synthesis [13], and animals ingesting DON may show symptoms of vomiting and feed refusal [14].

Enzymes known as trichothecene 3-*O*-acetyltransferases have the ability to modify DON by converting it to an acetylated derivative [15]. These enzymes are also produced by fungi in the genus *Fusarium*, and are encoded by the genes *TRI101 *or *TRI201 *[16,17]. The enzymatic modification involves the attachment of an acetyl group to the C-3 hydroxyl moiety of the trichothecene molecule [15], forming the derivative 3-acetyldeoxynivalenol (3ADON) [18]. Expression of *TRI101 *has been shown to reduce the phytotoxic effects of trichothecenes in tobacco and rice [19,20], and to decrease the inhibitory effects of trichothecenes on the growth of *Saccharomyces pombe *[18] and *Chlamydomonas reinnardtii *[21]. *In vitro *assays have shown DON to be more inhibitive than 3ADON of protein translation in rabbit reticulocytes [18], of DNA synthesis in mouse 3T3 fibroblasts [22] and of proliferation of murine lymphocytes [23]. However, 3ADON was only 1.4 times less toxic than DON in mice based on 50% lethal dose (LD_50_) values [24,25]. Although the difference in toxicity between DON and 3ADON *in vivo *is small, toxicology data in animals are limited [26], and to our knowledge, no alternative DON modification strategies with the potential to reduce toxicity are currently available.

Recently, seven trichothecene 3-*O*-acetyltransferases were evaluated for their ability to modify the mycotoxin DON [17]. In this study, we tested the hypothesis that two of these enzymes (FgTRI101 and FfTRI201) would reduce DON in DDGS resulting from a series of small-scale barley ethanol fermentations. To our knowledge, this is the first detailed report of yeast expressing a DON modification enzyme during barley ethanol fermentation, and provides a basis for evaluating novel detoxification enzymes such as DON de-epoxide hydrolases to reduce DON in DDGS [22,27].

## Methods

### Yeasts, enzymes and barley genotypes

#### Yeast strains

We used two yeast strains in this study: a commercial alcohol yeast (Dry Ethanol Red^®^; Fermentis, Marcq-en-Baroeul, France), which was used as a representative yeast strain for industrial ethanol production, and the *S. cerevisiae *strain RW2802 (PDR5 *leu2 ura3-52 met5*; kindly provided by Dr J. Golin, The Catholic University, Washington, DC, USA). RW2802 was used either as untransformed (control) or transformed with *FgTRI101 *or *FfTRI201*, as described below. The media used for culturing wild-type and transformed RW2802 have been described previously [17].

#### Acetyltransferases

**Figure 1:**
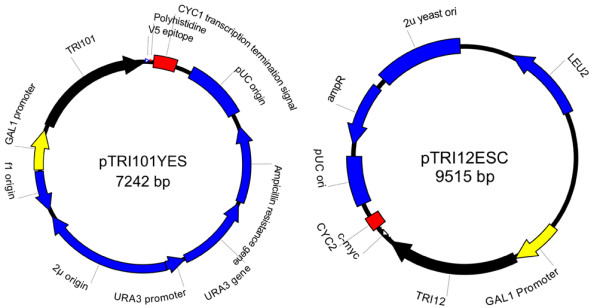
**Two vectors transformed into yeast strain RW2802 for barley fermentation assays**. (Left) The plasmid pTRI101YES contains either the *FgTRI101 or FfTRI201 *gene; (right) he pTRI12ESC vector is derived from pESC-LEU, and contains the *Fusarium sporotrichioides TRI12 *gene.

Gene isolation, cloning and expression of *FgTRI101, FfTRI201 *and *FsTRI12 *(a trichothecene efflux pump) were conducted as described previously [17]. The two vectors transformed into RW2802 for fermentation assays (Figure 1) were created using SeqBuilder (Lasergene version 8.1.1; DNAStar, Madison, WI, USA). Plasmid pTRI101YES contained either the *FgTRI101 *or the *FfTRI201 *gene (Figure 1). The pTRI12ESC vector was derived from pESC-LEU, and contained the *F. sporotrichioides TRI12 *gene (FsTRI12) (Figure 1).

#### Barley cultivars and experimental lines

In this study, we used both hulled and hulless winter barley: the hulled cultivars Price and Thoroughbred and the experimental hulled barley line VA04B-125, plus the hulless cultivar Eve and the experimental hulless line VA06H-25. These five types of barley were planted in a randomized complete block in mist-irrigated nurseries (Mt Holly, VA, USA). Plots 1.5 m × 13.4 m in size were used to produce sufficient material for analysis of DON concentrations in barley grain, barley mash and DDGS. Corn (*Zea mays*) kernels colonized with Virginia strains of *F. graminearum *were applied to plots at the boot stage of growth to encourage infection and DON contamination in the harvested grain. Grain was harvested in summer 2010 using a small research plot combine.

### Preparation and fermentation of the barley mashes

#### Eve, Price, Thoroughbred and VA06H-25

The following method for making barley mash was based on the enhanced dry grind enzymatic (EDGE) process developed by Nghiem *et al*. [28], and was used for all of the genotypes (preparation of the mash for VA04B-125 had some modifications, described later). Small samples of barley kernels (2000 g) were cleaned using a dockage tester (Carter Day International, Minneapolis, MN, USA) and ground to a particle size of 1 mm (Model 1 Wiley mill; Thomas Scientific, Swedesboro, NJ, USA). Two mashes of 1250 g were prepared, each containing 20% w/w dry solids. Deionized (DI) water (mash 1) or 10% w/w galactose solution (mash 2) was added to the ground grain to reach a final mass of 1250 g, and the pH was adjusted to 5.2 with 5 mol/l sulfuric acid. Two enzymes, a β-glucanase (OPTIMASH BG; Genencor, Palo Alto, CA, USA) and an α-amylase (SPEZYME XTRA; Genencor) were then added at 29.6 μl (0.13 kg/ton dry solids) and 68.2 μl (0.30 kg/ton dry solids), respectively. Liquefaction was carried out at 90°C for 2 hours in an oil bath with mechanical stirring. During liquefaction, small volumes of DI water were added to compensate for water loss due to evaporation. After 2 hours, the mash was cooled in an ice-water bath to a temperature of 32°C. Once cooled, the mass of the mash was adjusted with DI water back to 1250 g. The pH was then adjusted to 4.5 with 5 mol/l sulfuric acid. A glucoamylase/protease mix (FERMENZYME L-400; Genencor) and a developmental β-glucosidase (Genencor) were added at 147.7 μl (0.65 kg/ton dry solids) and 138.6 μl (0.61 kg/ton dry solids), respectively. To provide a nitrogen source, 0.5 g of urea was added to achieve a final concentration of 400 mg/l.

Fermentation was then carried out in 250 ml shake flasks containing 100 g each of the appropriate mash. Of the nine flasks, three were designated for each strain of yeast: Dry Ethanol Red, RW2802, and RW2802 transformed with FgTRI101/FsTRI12. Each flask was inoculated with the appropriate yeast strain, and placed in a shaking incubator set at a speed of 200 rpm and a temperature of 30°C for 66 hours. The Dry Ethanol Red yeast was rehydrated in DI water at 5% w/w, and 0.75 ml of this slurry was added to each designated flask. For the untransformed and transformed yeast strain RW2802 inocula, 100 ml liquid cultures were grown for two days at 30°C in a shaking incubator set at 200 rpm. Cultures of RW2802 and the transformed yeast cells were then separated by centrifugation at 1500 *g *for 5 minutes. The supernatants were discarded, and the cell pellets were resuspended in 2 ml of DI water (final optical density at 600 nm (OD_600_) was approximately 15.2). A 1.0 ml aliquot of the appropriate liquid culture was added to each designated flask.

### VA04B-125 mashes

In a separate experiment to test the acetylation levels of two different acetyltransferases (FgTRI101 and FfTRI201), we used ground grain from hulled barley line VA04B-125. Mashes of the VA04B-125 hulled barley line were prepared using the same procedure described above for the other four types of barley, with the following modifications. Two 1500 g mashes were prepared. β-glucanase and α-amylase were added at 35.5 μl (0.13 kg/ton dry solids) and 81.8 μl (0.30 kg/ton dry solids), respectively. The glucoamylase/protease mix and β-glucosidase were added at 177.4 μl (0.65 kg/ton dry solids) and 166.4 μl (0.61 kg/ton dry solids), respectively, then 0.6 g urea was added. Twelve small-scale fermentations were performed, of which three were designated for each of the four yeast strains. These included Dry Ethanol Red, untransformed RW2802, RW2802 transformed with FgTRI101/FsTRI12 and RW2802 transformed with FfTRI201/FsTRI12. Fermentations were carried out for 71 hours. Cultures of untransformed and transformed yeast strain RW2802 were separated by centrifugation. The supernatants were discarded, and the cell pellets were resuspended in 3 ml of DI water (OD_600 _approximately 7.0). A 1.0 ml aliquot of liquid culture was added to each designated flask. A summary of the experiments described in this section is provided in Table 1.

**Table 1:** Combination of Virginia barley line/cultivar, yeast strain and treatment for each mash^1 ^prepared for deoxynivalenol (DON) modification

Barley line/cultivar	Yeast strain	Flasks, n	Treatment
VA06H-25^2^	Dry Ethanol Red	3	No galactose
	RW2802	3	No galactose
	RW2802 FgTRI101/FsTRI12	3	No galactose
	
	Dry Ethanol Red	3	Galactose
	RW2802	3	Galactose
	RW2802 FgTRI101/FsTRI12	3	Galactose

VA04B-125^3^	Dry Ethanol Red	3	No galactose
	RW2802	3	No galactose
	RW2802 FgTRI101/FsTRI12	3	No galactose
	RW2802 FfTRI201/FsTRI12	3	No galactose
	
	Dry Ethanol Red	3	Galactose
	RW2802	3	Galactose
	RW2802 FgTRI101/FsTRI12	3	Galactose
	RW2802 FfTRI201/FsTRI12	3	Galactose

Thoroughbred^3^	Dry Ethanol Red	3	No galactose
	RW2802	3	No galactose
	RW2802 FgTRI101/FsTRI12	3	No galactose
	
	Dry Ethanol Red	3	Galactose
	RW2802	3	Galactose
	RW2802 FgTRI101/FsTRI12	3	Galactose

Price^3^	Dry Ethanol Red	3	No galactose
	RW2802	3	No galactose
	RW2802 FgTRI101/FsTRI12	3	No galactose
	
	Dry Ethanol Red	3	Galactose
	RW2802	3	Galactose
	RW2802 FgTRI101/FsTRI12	3	Galactose

Eve^2^	Dry Ethanol Red	3	No galactose
	RW2802	3	No galactose
	RW2802 FgTRI101/FsTRI12	3	No galactose
	
	Dry Ethanol Red	3	Galactose
	RW2802	3	Galactose
	RW2802 FgTRI101/FsTRI12	3	Galactose

^1^For each barley genotype, three replicate mashes were prepared for each yeast strain, to give a total of 96 mashes. Mashes were prepared using either deionized water or 10% w/w galactose solution.

^2^Hulless genotype.

^3^Hulled genotype.

### Extraction of trichothecene mycotoxins from ground barley grain, barley mash and distillers dried grains with solubles

Grains from all five barley genotypes (VA06H-25, VA04B-125, Thoroughbred, Price and Eve) were ground in a mill as described above, and mycotoxin extractions were performed on 1 g subsamples. Each subsample was combined with 8 ml of extraction solvent (86% v/v acetonitrile in DI water) in a capped polypropylene tube, and placed on a shaker at 200 rpm for 1 hour at room temperature (approximately 25°C). DON was detected and quantified using gas chromatography/mass spectrometry (GC/MS) (see below).

For the fermentation mashes, subsamples of 1 mL (weighing about 1 g) were taken at 0, 20, 44 and 66 hours, except in the case of VA04B-125, for which samples were taken at 0, 23, 47 and 71 hours. Each mash subsample was added to 7 ml of extraction solvent (described above). The mash sample/solvent mixtures were placed on a shaker at 200 rpm for 1 hour at room temperature.

At the end of fermentation, the entire contents of the experimental flasks were transferred into aluminum weighing pans, and dried in an oven at 55°C for 4 days. The collected DDGS was ground in a coffee grinder (Hamilton Beach, Model 80365, Southern Pines, NC, USA) positioned at the 'espresso' and '12 cup' setting. The ground DDGS samples weighing 1 g were added to 8 ml of extraction solvent and shaken at 200 rpm for 1 hour at room temperature. The mass of DON and 3ADON in each DDGS sample was determined by multiplying the concentration (ppm) by the mass of each corresponding DDGS sample.

Extraction solvents from the mash and DDGS, containing trichothecene mycotoxins, were passed through a clean-up column composed of a 1 g mixture of C18 (VWR, Radnor, PA, USA) and aluminum oxide (Sigma-Aldrich, St. Louis, MO, USA) at a 1:3 ratio. A 2 ml aliquot of eluent was transferred to a glass test tube, and evaporated to dryness using a nitrogen evaporator set at 55°C, then 100 μl of the derivatization agent n-trimethylsilylimidazole (TMSI; Sigma-Aldrich, St. Louis, MO, USA) was added to the dried samples. After 30 minutes, 500 μl of isooctane containing 5 ppm of the chlorinated hydrocarbon mirex (Sigma-Aldrich, St. Louis, MO, USA) was added to each tube, followed by 500 μl of water to quench the reaction. Samples were mixed by vortex for 10 seconds, and 150 μl of the isooctane/mirex supernatant was removed and transferred to chromatography vials for GC/MS analysis. The GC/MS detected and quantified DON and 3ADON in ppm. The percentage DON conversion (DON to 3ADON) was determined by calculating the percentage of 3ADON concentration in relation to total toxin in the subsample (DON + 3ADON). The concentration of DON and 3ADON and the percentage conversion values are reported as means ± standard error of the mean (SEM).

### Gas chromatography/mass spectrophotometry analysis

GC/MS analysis was conducted using a GC/MS system (6890/5975; Agilent Technologies, Santa Clara, CA, USA) operating in selected ion monitoring (SIM) mode as described previously [17]. Mirex was used as an internal control at 0.5 ppm. SIM mode detected DON and 3ADON target ions at a mass:charge ratio of 512 and 392 respectively, with reference ions at 422 and 497 for DON and a reference ion at 467 for 3ADON. SIM mode detected mirex target ions at a mass:charge ratio of 272 with reference ions at 276 and 237. DON and 3ADON were quantified in the samples using a quadratic regression model using pure DON and 3ADON standards (Biopure, Tulin, Austria) at concentrations of 0.5, 1.0, 5, 10 and 30 ppm.

### Protein extraction from VA04B-125 mashes

Protein extraction for the barley-mash subsamples was conducted based on the method of Kushnirov [29] for western blot analysis. A subsample of 1 g was taken at the end of fermentation (71 hours) from mashes containing 1) transformed RW2802 expressing FgTRI101, 2) transformed RW2802 expressing FfTRI201, and 3) untransformed RW2802. Subsamples were separated by centrifugation at 1500 *g *for 5 min. The supernatant was removed, and the mash pellet was resuspended in a mixture of 500 μl DI water and 500 μl of 0.2 mol/l NaOH, and held at room temperature for 5 minutes. After incubation at room temperature, the yeast cells were recovered by centrifugation, resuspended in 250 μl of SDS sample buffer [29], and boiled for 3 minutes. Samples (4 μl) of supernatant were loaded onto a 12% acrylamide SDS-PAGE gel, and run at 150 V for 1 hour, with a standard (Precision Plus Protein Dual Color; Bio-Rad, Hercules, CA, USA) used to determine protein size. After separation, protein transfer to a nitrocellulose membrane (Bio-Rad) was conducted in a transfer chamber at 34 mA for 1 hour at room temperature. The transfer buffer was composed of 25 mmol/l Tris, 190 mmol/l glycine, 2% SDS and 20% liquid chromatography-MS-grade methanol. The membrane was then blocked in 7% nonfat dry milk in Tris-buffered saline with Tween (TBST; 10 mmol/l Tris pH 8, 150 mmol/l NaCl, 0.05% Tween 20) for 1 hour at room temperature. The membrane was incubated with rabbit anti-FsTri101 primary antibody for 1 hour in 7% milk-TBST (1:5,000). After incubation with the primary antibody, the membrane was washed three times with 7% milk-TBST for 15 minutes each time. The membrane was incubated with the secondary antibody (alkaline phosphatase-conjugated anti-rabbit) for 1 hour in 7% milk-TBST solution (1:10,000). The membrane was washed in TBST three times for 15 min each time, and then washed in TBS (without Tween 20) once for 15 minutes. The membrane was incubated for 3 minutes with the substrate (Lumi-Phos WB; Fisher Scientific, Pittsburgh, PA, USA) at a volume of 0.125 ml for every cm^2 ^of membrane. X-ray film was exposed to the membrane for 5 minutes and developed. FgTRI101 and FfTRI201 were purified from *Escherichia coli *as described previously,[17] and were used as reference controls for the western blot.

### Ethanol and sugar quantification

For each subsample of mash taken during the time course study, the concentrations of ethanol, glucose and galactose were measured using an HPLC system (1200 Series; Agilent) equipped with a refractive index detector and a column (Aminex HPX-87H; Bio-Rad) with a guard column operating at 65°C. The mobile phase was 5 mmol/l H_2_SO_4 _pumped at a flow rate of 0.6 ml/min. An additional 1 ml mash sample was removed and separated by centrifugation at 12,000 rpm for 5 minutes. The supernatants were passed through a 0.2 μm filter (TITAN; Fisher Scientific, Pittsburg, PA, USA), and stored in the freezer until HPLC analysis.

Theoretical ethanol yields based on the total starch plus β-glucans were calculated as described previously [28]. In experiments using Dry Ethanol Red yeast and 10% w/w galactose solution for mashing, galactose was also included in the total available fermentable substrates because the yeast strain used was also capable of metabolizing this sugar.

### Composition of distillers dried grains with solubles

Compositional analysis of DDGS samples were conducted as described previously [28].

### Statistical analyses

All comparisons were performed using the statistical program JMP (version 9.0.0; SAS Institute Inc., Cary, NC, USA). To measure significant differences, analyses of variance (ANOVA) were performed. If a significant difference (*P *< 0.05) was found with ANOVA, then Tukey-Kramer's honestly significant difference (HSD) *post hoc *test was performed.

## Results

**Figure 2:**
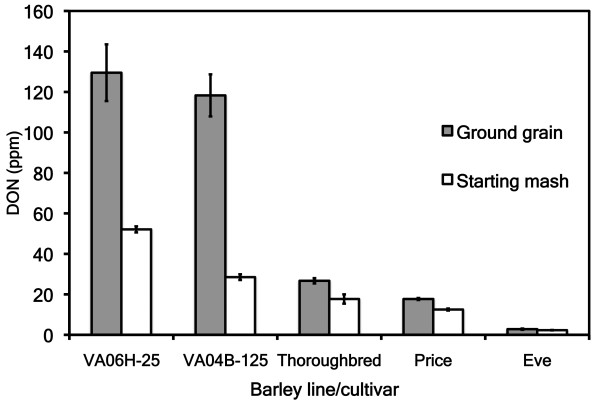
**Deoxynivalenol (DON) concentrations in ground grain and starting (0 hours) mash (with galactose)**. DON was diluted upon creation of the mash by 1.2 to 4.2 times.

Data were analyzed from 96 small-scale barley-fermentation mashes prepared from five barley genotypes (VA06H-25, VA04B-125, Thoroughbred, Price and Eve). DON levels (mean ± SEM) in the ground grain were 129.5 ± 14.0, 118.3 ± 10.4, 26.7 ± 1.3, 17.7 ± 0.5 and 2.8 ± 0.3 ppm, respectively (Figure 2). Two mashes, one with galactose and one without galactose, were prepared for each barley crop using ground grain (Table 1).

### Deoxynivalenol/3-acetyldeoxynivalenol concentrations in barley mashes with galactose

**Figure 3:**
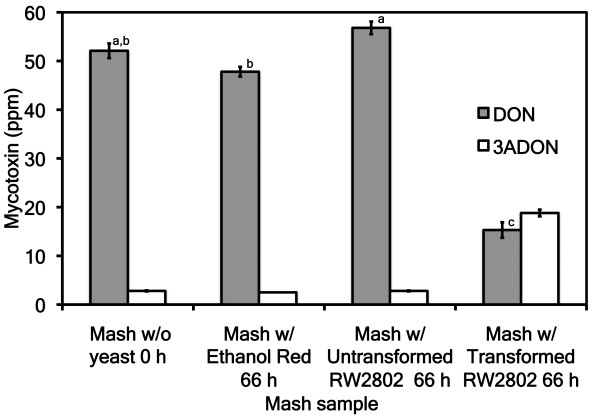
**Deoxynivalenol (DON) and 3-acetyldeoxynivalenol (3ADON) concentrations for VA06H-25 hulless barley in fermentations containing galactose**. Bars not connected by the same letter are significantly different. The concentration of DON in the mash with transformed RW2802 was significantly lower than both the starting mash without yeast and the mashes containing Ethanol Red and untransformed RW2802 yeast (*P *< 0.05).

Addition of galactose induced FgTRI101/FsTRI12 or FfTRI201/FsTRI12 expression in the transformed yeast strain RW2802. Upon preparation of the mash (0 hours), DON levels in the dry grain were diluted with the addition of 10% galactose solution (Figure 2). At 0 hours of fermentation (no yeast), DON levels were 52.1 ± 1.5 ppm (VA06H-25), 28.5 ± 1.4 ppm (VA04B-125), 17.7 ± 2.3 ppm (Thoroughbred), 12.5 ± 0.5 ppm (Price) and 2.3 ± 0.1 ppm (Eve) (Figure 2). At the end of the fermentation, DON concentrations were reduced in all mashes containing transformed yeast, but were significantly reduced only in the VA06H-25 mashes (*P *< 0.01) (Figure 3). The concentration of DON in VA06H-25 after 66 hours of fermentation was 15.3 ± 1.6 ppm (transformed RW2802), 56.8 ± 1.3 ppm (untransformed RW2802) and 47.8 ± 1.0 ppm (Ethanol Red) (Figure 3). The concentration of 3ADON in VA06H-25 after 66 hours of fermentation was 18.8 ± 0.7, 2.8 ± 0.1 and 2.5 ± 0.0 ppm for transformed RW2802, untransformed RW2802 and Ethanol Red, respectively (Figure 3).

### Percentage deoxynivalenol conversion in Eve, Price, Thoroughbred and VA06H-25 mashes

**Figure 4:**
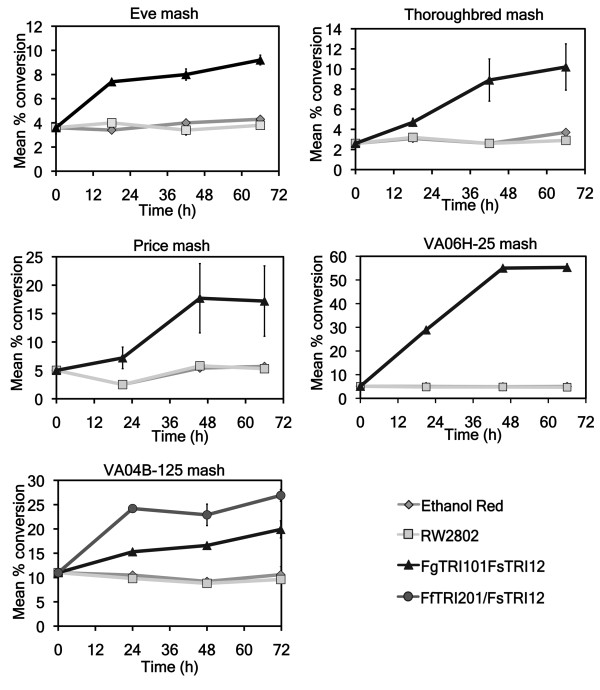
**Mean percentage conversion of deoxynivalenol (DON) to 3-acetyldeoxynivalenol (3ADON) for barley fermentations containing galactose**. For each time point, the SEM is based on three replications. Note that the *y *axes have been scaled for each of the barley genotypes. Study was conducted for 66 hours and, subsamples were taken at 0, 20, 44 and 66 hours. For the comparison between the two trichothecene 3-*O*-acetyltransferases, the VA04B-125 mash samples were taken at 0 h, 23 h, 47 h and 71 hours. Mycotoxin extractions were performed on all subsamples and analyzed by gas chromatography/mass spectrometry.

Mash subsamples were collected over a period of 66 hours from all mashes containing galactose. Subsamples were taken at 0, 20, 44 and 66 hours. In 20 hours, mean conversion levels ranged from 4.7 ± 0.4% (Thoroughbred) to 28.9 ± 1.0% (VA06H-25). At 44 hours, the mean conversion ranged from 8.0 ± 0.5% (Eve) to 55.0 ± 1.2% (VA06H-25) for mashes with transformed yeast. The end of fermentation with transformed yeast yielded mean conversions ranging from 9.2 ± 0.7% (Eve) to 55.3 ± 1.8% (VA06H-25). For each time point after 0 hours, mashes with transformed yeast had significantly higher conversion values than those with untransformed yeast strains (*P *< 0.05 for pairwise comparisons conducted within each barley crop) (Figure 4). For untransformed yeast strains, the highest conversion at the end of the assay was 5.7 ± 0.1% (Price, 66 hours) (Figure 4).

### Percentage deoxynivalenol conversion in VA04B-125 mashes

In a separate experiment, the acetylation levels of two different acetyltransferases (FgTRI101 and FfTRI201) were compared using ground grain from the hulled barley line VA04B-125. Subsamples were collected at 0, 23, 47 and 71 hours. In VA04B-125 mashes, FfTRI201 produced a greater reduction in DON and an increased conversion of DON to 3ADON compared with FgTRI101 (Figure 4). DON conversion levels at the end of the assay for mashes with Ethanol Red and untransformed RW2802 were 10.6 ± 0.9% and 9.6 ± 0.4%, respectively, whereas RW2802 expressing FgTRI101 or FfTRI201 had levels of 19.9 ± 1.0% and 26.9 ± 0.7%, respectively (Figure 4).

**Figure 5:**
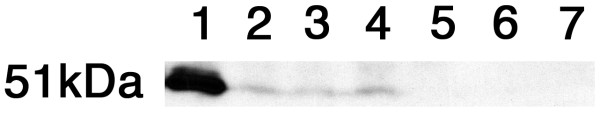
**Western blot of the TRI201 enzyme from *Fusarium fujikuroi *(FfTRI201) extracted from VA04B-125 mashes**. Mash samples weighing 1 g were collected at the end of fermentation (71 hours), and protein extractions were performed. Mashes containing untransformed RW2802 yeast were used as a negative control. For analysis, 4 μl of each extract were loaded onto a 12% acrylamide-SDS-PAGE gel and run at 150 V for 1 hour. A protein standard (Precision Plus Protein Dual Color Standard) was used to determine protein size. The western blot was probed with rabbit anti-FsTRI101 primary antibody, and the probe detected with alkaline phosphatase-conjugated anti-rabbit antibody. Lane 1: purified FfTRI201 from *Escherichia coli*; lane 2: FfTRI201 from mash 1 containing transformed yeast; lane 3 FfTRI201 from mash 2 containing transformed yeast; lane 4: FfTRI201 from mash 3 containing transformed yeast; lane 5: mash 1 containing untransformed RW2802; lane 6: mash 2 containing untransformed RW2802; and lane 7: mash 3 containing untransformed RW2802.

Protein extractions were conducted on mashes to reveal the levels of FgTRI101 and FfTRI201. Western blot analysis detected the presence of FfTRI201 protein in all three mashes with *FfTRI201*-transformed RW2802 (Figure 5). No protein was detected in untransformed RW2802 mashes (Figure 5). Protein extractions conducted on mashes with *FgTRI101*-transformed yeast did not yield FgTRI101 protein in our western blot analysis, probably because FgTRI101 was below detection levels (data not shown).

### Deoxynivalenol/3-acetyldeoxynivalenol in Eve, Price, Thoroughbred and VA06H-25 distillers dried grains with solubles

The concentration of DON in DDGS was 1.6 to 8.2 times higher than the original ground grain used to prepare the mashes (Table 2). The mass of DON in DDGS samples was lower for all mashes with the transformed RW2802 yeast compared with mashes containing either the untransformed RW2802 or Ethanol Red (Table 3). In the recovered DDGS samples, the mass of DON in DDGS from fermentations with untransformed RW2802 ranged from 178.4 ± 8.2 μg (Eve) to 2496.2 ± 47.6 μg (VA06H-25), whereas it ranged from 157.2 ± 7.2 μg (Eve) to 1098.7 ± 39.4 μg (VA06H-25) from fermentations with transformed RW2802 (Table 3). This reduction was significant for Price and Thoroughbred, the hulled barley cultivars (*P <*0.05) (Table 3). The DON in DDGS from the hulless line VA06H-25 was significantly lower when derived from mashes with transformed RW2802 than with either untransformed RW2802 or Ethanol Red *P <*0.05) (Table 3). The mass of 3ADON was significantly higher in all DDGS samples with transformed RW2802 than in DDGS samples containing untransformed RW2802 or Ethanol Red (Table 3). The largest reduction of DON using transformed yeast, was seen in VA06H-25 DDGS (DON:3ADON ratio of 0.4 ± 0.0), and the smallest in Thoroughbred DDGS (2.9 ± 0.8) (Table 3).

**Table 2:** Comparison of deoxynivalenol (DON) concentration in ground grain versus distillers dried grains with solubles (DDGS) from fermentation with Ethanol Red

Barley line/cultivar	Ground grain, mean ppm ± SEM	Ethanol Red DDGS (no galactose), mean ppm ± SEM	Times DON concentrated in DDGS^1^
VA06H-25^2^	129.5 ± 14.0	212.9 ± 3.0	1.6

VA04B-125^3^	118.3 ± 10.4	207.1 ± 3.0	1.8

Thoroughbred^3^	26.7 ± 1.3	130.9 ± 2.4	4.9

Price^3^	17.7 ± 0.5	99.4 ± 3.5	5.6

Eve^2^	2.8 ± 0.3	23.0 ± 0.3	8.2

^1^The concentration of DON in the DDGS was 1.6 to 8.2 times that in the original starting grain.

^2^Hulless genotype.

^3^Hulled genotype.

**Table 3:** Mass of deoxynivalenol (DON) and 3-acetyldeoxynivalenol (3ADON) in distillers dried grains with solubles (DDGS) derived from mashes amended with galactose

Barley line/cultivar	Mycotoxin	Mass^1 ^of mycotoxin^2 ^(mean μg ± SEM) in DDGS with:		
		
		Ethanol Red	Untransformed RW2802	Transformed RW2802
VA06H-25^3^	DON	1854.6 ± 55.8^a^	2496.2 ± 47.6^b^	1098.7 ± 39.4^c^
	3ADON	689.2 ± 12.4^a^	573.8 ± 27.5^a^	2500.2 ± 40.7^b^
	Ratio	2.7 ± 0.1^a^	4.4 ± 0.1^b^	0.4 ± 0.0^c^

VA04B-125^4^	DON	2206.2 ± 8.1^a^	1951.8 ± 34.4^b^	1775.1 ± 36.1^c,5^
	3ADON	233.8 ± 3.1^a^	229.1 ± 6.1^a^	560.1 ± 10.7^b,5 ^
	Ratio	9.4 ± 0.1^a^	8.5 ± 0.1^b^	3.2 ± 0.1^c,5^

Thoroughbred^4^	DON	1100.0 ± 4.2^a, b^	1165.7 ± 55.6^a^	1001.5 ± 33.2^b^
	3ADON	183.4 ± 1.5^a^	130.2 ± 7.5^b^	369.0 ± 7.5^c^
	Ratio	6.0 ± 0.1^a^	9.0 ± 0.1^b^	2.9 ± 0.5^c^

Price^4^	DON	974.4 ± 8.1^a, b^	1090.4 ± 35.4^a^	785.2 ± 86.7^b^
	3ADON	123.6 ± 1.6^a^	113.0 ± 5.2^a^	391.0 ± 123.3^b^
	Ratio	7.9 ± 0.1^a^	9.7 ± 0.1^b^	2.5 ± 0.8^c^

Eve^3^	DON	195.0 ± 3.7^a^	178.4 ± 8.2^a, b^	157.2 ± 7.2^b^
	3ADON	36.2 ± 2.0^a^	22.6 ± 4.0^b^	54.2 ± 2.1^c^
	Ratio	5.4 ± 0.4^a^	8.7 ± 2.3^a^	2.9 ± 0.3^b^

^1^Mass values of deoxynivalenol not connected by the same letter across rows are significantly different (*P <*0.05) from each other.

^2^Mycotoxin extraction was performed on 1 g subsamples of DDGS and analyzed by gas chromatography/mass spectrometry.

^3^Hulless genotype.

^4^Hulled genotype.

^5^DON and 3ADON mass from transformed yeast expressing FfTRI201.

### Deoxynivalenol/3-acetyldeoxynivalenol in VA04B-125 distillers dried grains with solubles

In VA04B-125 DDGS, the mean concentration of DON was concentrated 1.8 times compared with the starting ground grain used to prepare the mashes (Table 2). In a comparison between FgTRI101 and FfTRI201 in DDGS derived from VA04B-125 mashes, FfTRI201 produced the greatest reduction in DON. Average DON levels were 1775.1 ± 36.1 μg (FfTRI201), 1845.7 ± 55.5 μg (FgTRI101), 1951.8 ± 34.4 μg (untransformed RW2802) and 2206.2 ± 8.1 μg (Ethanol Red) for the VA04B-125 DDGS (Table 3). DDGS from fermentations with transformed RW2802 had DON levels that were significantly lower than those with Ethanol Red or untransformed RW2802 (*P *< 0.05) (Table 3). The 3ADON mean mass for DDGS with transformed RW2802 was significantly higher than that for DDGS with Ethanol Red or untransformed RW2802 (*P *< 0.05) (Table 3).

### Sugar consumption and ethanol yields

Unlike RW2802, the Ethanol Red yeast consumed galactose (Table 4), leading to significantly higher ethanol concentrations in galactose-containing mashes compared with galactose-free mashes *(P *< 0.05) (Table 4). In galactose-free mashes, ethanol yields ranged from 67.45% (VA06H-25 with untransformed RW2802) to 91.41% (VA04B-125 with Ethanol Red) (Table 4), whereas in galactose-containing mashes, ethanol yields ranged from 36.06% (Thoroughbred with untransformed RW2802) to 94.74% (VA04B-125 with Ethanol Red) (Table 4). In a comparison between galactose-free mashes, RW2802 produced significantly less ethanol than did Ethanol Red *(P *< 0.01) (Table 4). Since transgene expression only occurred by galactose induction, we examined whether ethanol yields were different between transformed and untransformed RW2802, not accounting for the type of barley. ANOVA showed that ethanol yields were not significantly different between untransformed RW2802 and transformed RW2802 expressing FgTRI101 or FsTRI12 (*P *= 0.23).

**Table 4:** Final average ethanol (% v/v) for mashes with Ethanol Red, untransformed RW2802 and transformed RW2802 yeasts

Barley line/cultivar	Yeast strain	No galactose	10% Galactose		Ethanol yields	
		
		Final ethanol, % v/v	Final ethanol, % v/v	Final galactose, % v/v	Without galactose. %	With galactose. %
VA06H-25^1^	Ethanol Red	9.84	14.43^3^	0.14	88.49	90.93
	Untransformed RW2802	7.50	7.40	8.37	67.45	46.63
	RW2802 transformed with FgTRI101	7.51	7.57	8.03	67.54	47.70

VA04B-125^2^	Ethanol Red	7.75	12.79^3^	0.44	91.41	94.74
	Untransformed RW2802	6.72	6.24	8.78	79.26	46.22
	RW2802 transformed with FgTRI101	7.63	7.68	8.84	90.00	56.89
	RW2802 transformed with FfTRI201	7.74	7.59	8.73	91.30	56.22

Thoroughbred^2^	Ethanol Red	9.43	14.26^3^	0.33	83.53	88.96
	Untransformed RW2802	8.48	5.78	8.90	75.11	36.06
	RW2802 transformed with FgTRI101	8.25	6.93	8.77	73.07	43.23

Price^2^	Ethanol Red	8.27	13.24^3^	0.39	87.42	92.01
	Untransformed RW2802	6.47	7.37	8.57	68.39	51.22
	RW2802 transformed with FgTRI101	7.93	7.07	8.83	83.83	49.13

Eve^1^	Ethanol Red	9.87	13.58^3^	1.72	85.60	83.62
	Untransformed RW2802	9.49	6.68	9.11	82.31	41.13
	RW2802 transformed with FgTRI101	9.24	6.19	8.94	80.14	38.12

^1^Hulless genotype.

^2^Hulled genotype.

^3^Using Dry Ethanol Red yeast in mashes with galactose resulted in significantly higher ethanol yields than from the same mash without galactose (*P <*0.05). Ethanol yields are also shown for other mashes with and without galactose.

### Composition of distillers dried grains with solubles

In a preliminary analysis of DDGS composition, DDGS samples from fermentation with transformed RW2802 yeast were found to be similar to those produced with commercial Ethanol Red yeast (data not shown). Differences in composition occurred only with mashes amended with galactose in which galactose and residual sugars in fermentations were not utilized to completion. RW2802 did not consume galactose during fermentations, causing components of its DDGS to be diluted compared with the Ethanol Red DDGS. For example, the DDGS from VA06H-25 fermented with Ethanol Red in the presence of galactose was composed of 24.43% protein, 31.27% neutral detergent fiber (NDF), 1.50% starch, 0.22% β-glucan and 5.06% crude fat, whereas the DDGS from the same barley line fermented with transformed RW2802 in the presence of galactose contained 13.33% protein, 14.32% NDF, 11.23% starch, 0.14% β-glucan and 2.41% crude fat, (data not shown).

## Discussion

The fungal plant pathogen *F. graminearum *produces trichothecene mycotoxins that may remain as a contaminant in barley DDGS after fuel ethanol production [30]. New cost-effective and commercially viable methods to reduce mycotoxin contamination in barley DDGS need to be developed and implemented. Our work has a direct relevance to commercial barley ethanol plants in the USA (such as the ethanol plant in Hopewell, Virginia) and in Europe. Preparation of the barley-mash dilutes mycotoxin levels from the ground grain through the addition of DI water or 10% galactose solution (Figure 2). Mycotoxin levels are then concentrated during the formation of DDGS (Table 2). DON is soluble in water [31], and therefore we would expect a mycotoxin dilution of approximately fourfold in the mash compared with the dry grain (all mashes in this study were 20% solids). However, not all DON may dissolve in water [32], and therefore increases in ground grain taken from the mash during subsampling may explain the smaller dilutions when the concentration of dry ground grain is compared with levels in the mash at the start of fermentation (Figure 2).

We found large reductions in DON via conversion (52.4% to 58.1%) during fermentation of the hulless barley line VA06H-25, which contained the highest levels of DON in its starting ground grain (Figure 4). This alone demonstrates the tremendous potential for commercial ethanol yeasts to be engineered to express enzymes that modify mycotoxins (such as trichothecene 3-*O*-acetyltransferases) during fermentation. In a recent study, seven different trichothecene 3-*O*-acetyltransferases transformed into the yeast strain RW2802 were analyzed for their ability to modify DON into 3ADON during a series of feeding assays [17]; conversion levels ranged from 50.5% to 100%, depending on the source of the acetyltransferase [17]. In our study, the enzyme FgTRI101 resulted in a 55.3% mean conversion of DON for the VA06H-25 (hulless barley line), but previous feeding assays with the same enzyme reported a reduction of 92.6% in yeast cultures [17]. There may be several reasons for the different levels of conversion in our barley ethanol fermentations compared with the previously published feeding assays. It is possible that 'pure' yeast cultures allow higher acetylation rates because of the greater accessibility to DON by the acetyltransferases. The complex matrix of proteins and sugars in barley mashes [33] might impede the ability of the acetyltransferases to interact with DON. The starting concentration of yeast might also play a role in determining DON acetylation rates; the OD_600 _of yeast inoculum for our hulled line (VA04B-125) was approximately half that of the inoculum for the hulless line (VA06H-25), and might have contributed to the differences in acetylation rates during fermentation between these two lines.

We compared the acetylation levels of two different acetyltransferases (FgTRI101 and FfTRI201) during fermentation, using ground grain from VA04B-125 (hulled barley). Previous work has shown that the enzyme FgTRI101 has a catalytic efficiency towards DON that is 9.2 times greater than that of FfTRI201, but FfTRI201 results in higher DON conversion levels than FgTRI101 likely because of its higher protein expression in yeast [17]. In our study, FfTRI201 converted more DON to 3ADON during fermentation than did FgTRI101 (Figure 4), and this was confirmed in the corresponding DDGS (data not shown). Western blot analyses of mashes containing VA04B-125 detected FfTRI201 in all three mashes tested, but FgTRI101 was not detected. Previous studies have reported that FfTRI201 is expressed at higher levels than FgTRI101 in yeast [17], which might explain why the FgTRI101 levels in the VA04B-125 mashes were below the limit of detection in our western blot.

In our fermentation assays, it is likely that glucose (repression) and galactose (induction) were competing for control of the *GAL1 *promoter (Figure 1), responsible for FgTRI101 and FfTRI201 expression, and therefore the expression of the acetyltransferases may not have been optimal in the fermentations. Alternative methods to induce protein expression (for example, using inducers other than galactose) may yield larger reductions in DON, especially in grain containing reduced amounts of DON (the substrate). Future studies could use promoters such as *CUP1 *[34] induced by copper (100 μmol/l Cu^2+^) [35,36]. The effect of copper on fermentation and DDGS production is unknown; however, addition of copper (30 mg/kg dry mass) to animal feed has been reported to suppress bacterial infections in the gut of swine [37]. Alternatively, for constitutive expression, the phosphoglycerate kinase promoter (*PGK1*) can be used, and requires no additional components [38].

Previous reports have indicated a threefold increase in the concentration of DON in DDGS relative to starting material [39]. In our study, DON concentrations in DDGS from Ethanol Red fermentations were about 1.6 to 8.2 times higher than in the starting ground grain (Table 2). Unexpectedly, ground grain from resistant genotypes (e.g., Eve), containing a low DON concentration, resulted in the corresponding DDGS having DON levels that were concentrated more than those in DDGS derived from ground grain with high DON levels (e.g., VA06H-25) (Table 2). It is possible that resistant genotypes harbor more masked DON (DON glucosides), through expression of a UDP-glucosyltransferase, [40,41] than do susceptible genotypes (which accumulate high levels of DON), which may be subsequently hydrolyzed by the yeast, causing DON to be released during fermentation [42]. This may help explain our results (Table 2) showing DON concentrating in DDGS relative to the ground grain, but this was not investigated further in the present study, and we were unable to calculate a proper mass balance to compare the masses of DON because of the subsampling of mashes during the course of the fermentation.

The reduction in total solid mass during fermentation (in which glucose is converted to ethanol and carbon dioxide), together with the loss of moisture during drying of the DDGS, increases the concentration of mycotoxins in DDGS. Because the laboratory yeast strain RW2802 does not consume galactose, the components (including DON) of its corresponding DDGS were diluted. Mycotoxin dilutions caused by galactose and other residuals (such as unreacted starch, oligosaccharides, maltose and glucose) remaining because of incomplete fermentation, made calculating the concentration of mycotoxins in the DDGS unreliable, and therefore a mass balance was used (Table 3). Fermentations containing yeast transformed with FgTRI101 or FfTRI201 reduced the mass of DON and increased the mass of 3ADON in all DDGS samples (Table 3). These enzymes are probably inactive in the DDGS because the thermostability values of these enzymes [17] are approximately 15°C lower than the temperature at which the DDGS was prepared.

Ethanol yields were greatest in mashes containing Ethanol Red and galactose. This industrial yeast strain was developed for fuel ethanol production and has the unique ability to utilize both galactose and glucose. In most yeast strains, galactose utilization is about one-third that of glucose [43]. The model (laboratory) yeast strain RW2802 does not have the ability to utilize galactose efficiently, thus in our experiments, ethanol yields for RW2802 were significantly lower in the presence of galactose. This is perhaps due to the energy cost on the yeast cells to synthesize enzymes in the Leloir pathway, which make up approximately 5% of all total cellular enzymes [44]. DON is a known protein synthesis inhibitor [13], but ethanol yields were not affected by DON in our fermentations.

Another approach to reduce DON in DDGS might be to add an exogenous trichothecene 3-*O*-acetyltransferase preparation to the mash at the start of fermentation. However, the amount of enzyme needed for this approach to be successful is presently unknown. Moreover, the enzyme stability may limit the effectiveness of this strategy [15], and no such preparation is commercially available at this time. Washing the grain [32] before fermentation can be implemented in order to reduce DON levels before mash preparation, in addition to DON modificiation during fermentation. Reduction of mycotoxins in fermentation mashes does not have to be limited to barley. This strategy could also be applied to other fuel ethanol crops such as corn, wheat and sugarcane. For example, in addition to deoxynivalenol, the mycotoxin zearalenone is another common contaminant of corn ethanol co-products [45], and a lactonohydrolase has been shown to decrease levels of zearalenone in spiked cultures of *Schizosaccharomyces pombe *and *E. coli *[46].

The EDGE process was developed as a new method for increasing ethanol yields from barley in a commercial setting to advance biofuels made from non-food feedstocks [28]. Employing yeast to express mycotoxin-detoxification genes represents a potential strategy to reduce mycotoxin levels in fuel ethanol co-products. However, a number of issues must be addressed before this process is commercialized. First, integrating a transgene into the yeast genome would be preferred over maintaining the gene on a plasmid (which generally requires selective conditions for plasmid propagation). Second, the composition of DDGS in future work using transformed yeast would need to be evaluated. Analysis of DDGS composition in this study showed that DDGS produced by transformed yeast was similar to DDGS produced by commercial yeast, except for the change in the concentration of components due to added galactose and residual sugars that were not utilized to completion. Third, the use of a transgenic yeast strain for fuel ethanol production will need to be accepted by policy makers and ethanol production facilities in order to be implemented on a commercial scale.

## Conclusions

When using transformed yeast expressing a trichothecene 3-*O*-acetyltransferase in small-scale barley fermentations, DON contaminating the ground grain was converted to 3ADON, and thereby the concentration of DON was reduced in DDGS. FfTRI201 resulted in higher acetylation levels than those resulting from FgTRI101 during fermentations in VA04B-125 mashes. In DDGS derived from mashes containing Ethanol Red, DON levels were concentrated 1.6 to 8.2 times compared with those in ground grain, depending on the barley line/cultivar used in the mash, but were reduced when transformed yeast expressing either FgTRI101 or FfTRI201 was used. Mashes with Ethanol Red yeast had higher ethanol yields than mashes with the laboratory yeast strain RW2802. In mashes with galactose, Ethanol Red was able to utilize this sugar for conversion into ethanol, whereas the galactose resulted in reduced ethanol yields with RW2802. To our knowledge, this is the first detailed report of yeast expressing a DON modification enzyme during barley ethanol fermentation, and provides a basis for evaluating novel detoxification enzymes such as DON de-epoxide hydrolases to reduce DON in DDGS in the future.

## Abbreviations

ANOVA: analysis of variance; 3ADON: 3-acetyldeoxynivalenol; DDGS: ]distillers dried grains with solubles; DI: deionized DON; deoxynivalenol; EDGE: enhanced dry grind enzymatic; FHB: *Fusarium *head blight; GC/MS: gas chromatography/mass spectrometry; HPLC: high performance liquid chromatography; OD: optical density; ppm: parts per million; rpm: rotations per minute; SDS: sodium dodecyl sulphate; SEM: standard error of the mean; SIM: select ion monitoring; TBST: Tris-buffered saline with Tween; TMSI: trimethylsilylimidazole.

## Competing interests

The authors declare that they have no competing interests.

## Authors' contributions

PAK was involved in the design of the experimental work, performed experiments and analytical work involving transgenic yeast, and was the lead writer on the manuscript. JM performed experiments and analytical work on ethanol and sugar quantification, and assisted in editing and writing the manuscript. NPN and KBH contributed to the design, helped coordinate experimental work, and assisted in editing and writing the manuscript. GB, WSB and CAG were involved in development of the barley lines/cultivars used in this study, and performed all the field work necessary to grow and harvest the barley, and provide the grain. DGS secured funding for the project, and helped design and coordinate the experiments and resulting data analyses. All authors read and approved the final manuscript.
